# Integrating radiomics into holomics for personalised oncology: from algorithms to bedside

**DOI:** 10.1186/s41747-019-0143-0

**Published:** 2020-02-07

**Authors:** Roberto Gatta, Adrien Depeursinge, Osman Ratib, Olivier Michielin, Antoine Leimgruber

**Affiliations:** 1 Personalised Analytic Oncology, Department of Oncology, Lausanne University Hospital, Lausanne, Switzerland; 2 University of Applied Sciences and Arts Western Switzerland (HES-SO), Sierre, Switzerland; 3 Service of Medical Imaging, Riviera-Chablais Hospital, Rennaz, Switzerland; 4 Department of Medical Imaging, Lausanne University Hospital, Lausanne, Switzerland

**Keywords:** Artificial intelligence, Holomics, Machine learning, Precision medicine, Radiomics

## Abstract

Radiomics, artificial intelligence, and deep learning figure amongst recent buzzwords in current medical imaging research and technological development. Analysis of medical big data in assessment and follow-up of personalised treatments has also become a major research topic in the area of precision medicine. In this review, current research trends in radiomics are analysed, from handcrafted radiomics feature extraction and statistical analysis to deep learning. Radiomics algorithms now include genomics and immunomics data to improve patient stratification and prediction of treatment response. Several applications have already shown conclusive results demonstrating the potential of including other “omics” data to existing imaging features. We also discuss further challenges of data harmonisation and management infrastructure to shed a light on the much-needed integration of radiomics and all other “omics” into clinical workflows. In particular, we point to the emerging paradigm shift in the implementation of big data infrastructures to facilitate databanks growth, data extraction and the development of expert software tools. Secured access, sharing, and integration of all health data, called “holomics”, will accelerate the revolution of personalised medicine and oncology as well as expand the role of imaging specialists.

## Key points

- Since 2012, radiomics algorithms have focused on lesion characterisation and response prediction in oncology.
- Research advances are expected in deep learning, data harmonisation, and communication.
- Radiogenomics and radioimmunomics, alone or in combination with other data, improve prediction accuracy.
- Radiomics is a component of all omics data (holomics) for personalised decision-making.
- This major evolution relies heavily on information technology and medical imaging specialists.

## Background

Radiomics is now a field of intense research after 7 years of an exponential growth in publications. Radiomics offers the perspective to augment human perception with the use of agnostic analyses [1] and even artificial intelligence with computer-assisted increase in productivity. Naturally, this trend has been met with great enthusiasm and even greater scepticism. As the field matures, numerous initiatives have led to the development of a growing number of software solutions, libraries, and algorithms.

**Fig. 1:**
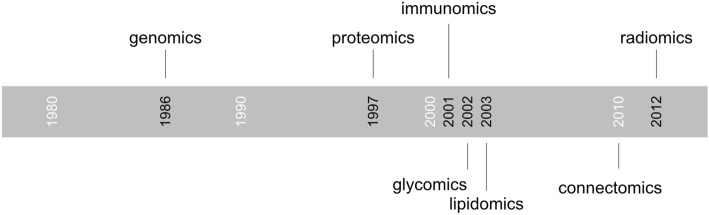
Timeline of the first occurrence of selected “omics” terms

Radiomics is of course not the first “omics” field, and genomics can be traced back to the late 1980s [2], followed by many others (Fig. 1). The suffix “omics” has long been used in life sciences to describe the techniques and data in a specific research field. In the case of radiomics, this suffix reflects the intent to comprehensively use the data provided in the medical images with mathematical and statistical approaches. As radiomics attempts to make its way into the clinical workflow, the use of imaging data alone is becoming insufficient. While there is no formal proof that clinical decisions based on the combination of imaging and other “omics” data have an impact on clinical outcome, there is no reason to limit the scope of algorithms. This approach is similar to clinical practice, where all available data about a particular patient and previous knowledge from other cases are aggregated in the decision process. Radiomics-based algorithms may hence combine genomics, immunomics or other clinical data to improve diagnostic accuracy.

In an ambitious paradigm shift, radiomics may further be integrated as a component of a comprehensive software system for clinical decision support combining all “omics” and clinical data into a “holomics” approach (“holo” meaning “whole” in classical Greek), similarly to systems biology in experimental research [3, 4].

**Fig. 2:**
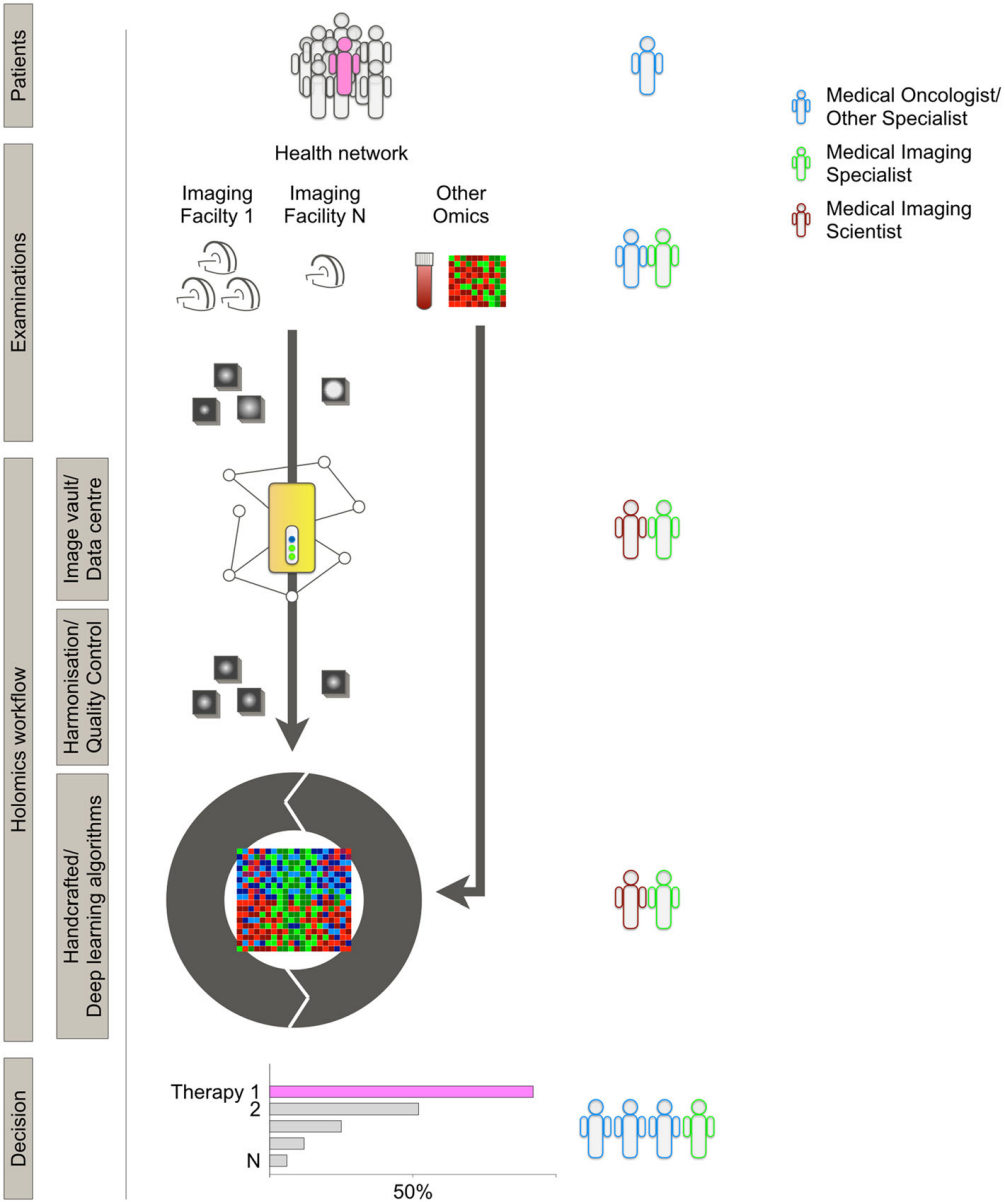
Illustration of the different elements of a model of integration of radiomics in a holomics-based clinical workflow. Patients: constant accumulation of patient data is used in a dynamic model. Examinations: images are produced with many protocols, machines, and facilities. Other "omics" data from blood tests, tumour samples, or clinical data are aggregated. Image vault/data centre: a collaborative, open-source, open data storage infrastructure guarantees secured ownership of data, and facilitated software development. Harmonisation/quality control: as for other omics, radiomics can only reach clinical practice and feed algorithms with harmonisation and quality control at each step. Decision: predictive information (rather than prognostic only) is provided to tumour boards or other specialist boards to provide support for decision

Widespread adoption of radiomics in the clinical decision process has been elusive despite a crucial need for prediction of treatment response and therapy management in the era of personalised medicine. With the development of large health networks and personalised health initiatives, the clinical data flow is rapidly expanding beyond what individual physicians can handle. Through their growing expertise in complex algorithms, big data management, and data harmonisation, medical and information technology imaging specialists are now key for the integration of radiomics and holomics in the clinical workflow of hospitals and health networks (Fig. 2).

This review summarises current research on radiomics with a focus on standardisation strategies developed to ensure reproducibility between studies, machines and centres. It also discusses biobanks and personalised medicine initiatives in order to illustrate the steps required for the integration of radiomics into holomics and, ultimately, into clinical workflows.

## Research trends in radiomics

### Publication trend

Radiomics aims to capture the informative content hidden in medical images, overcoming the limitations of the human eyes and human cognitive patterns: the rationale is that medical images (anatomical, functional, or metabolic) can carry information about the physiological response to cancer and therapy stress [1]. The wealth of relevant information provided by medical images is key in decision-making and follow-up of treatment response. The ability to extract more quantitative data from medical images will reinforce the position of medical imaging in clinical decision-making and patient management.

**Fig. 3:**
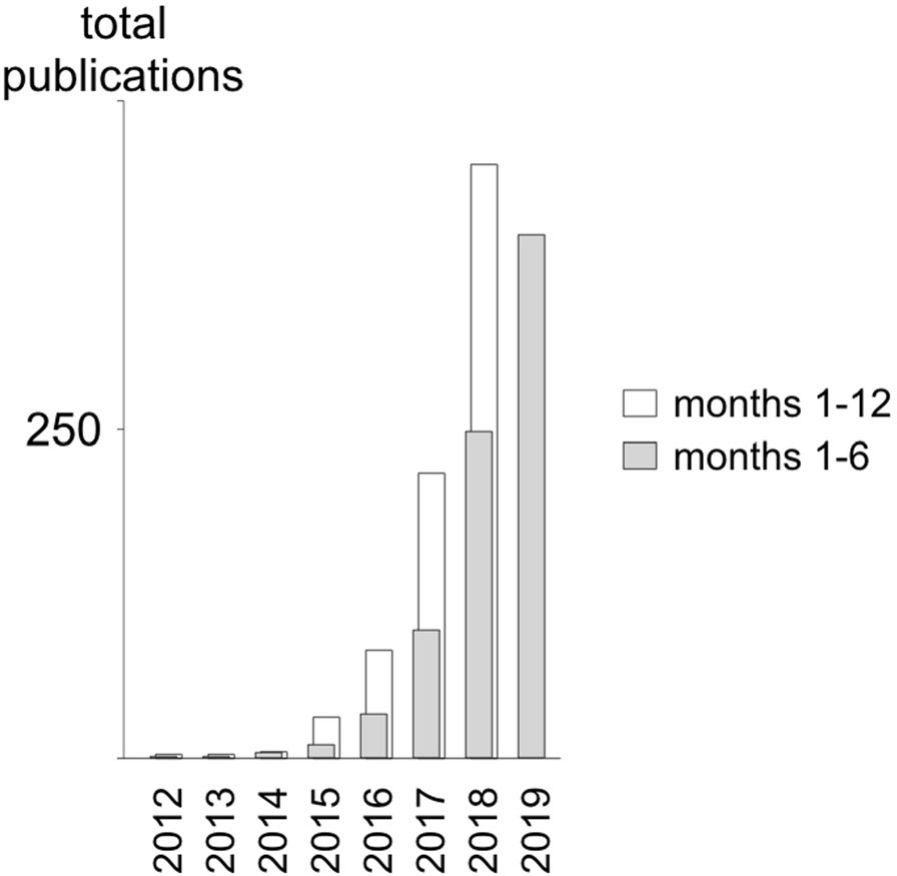
Timeline of the occurrence of radiomics publications from PubMed title and abstract search shows exponential growth since 2012 with a slowdown in early 2019

Medical and information technology imaging specialists have produced a number of algorithms, statistical analyses, and models, fuelling an exponential growth in the number of publications since 2012 (Fig. 3, Additional file 1: Table S1). These have gradually become more complex and study more diverse pathologies, modalities, and applications.

**Fig. 4:**
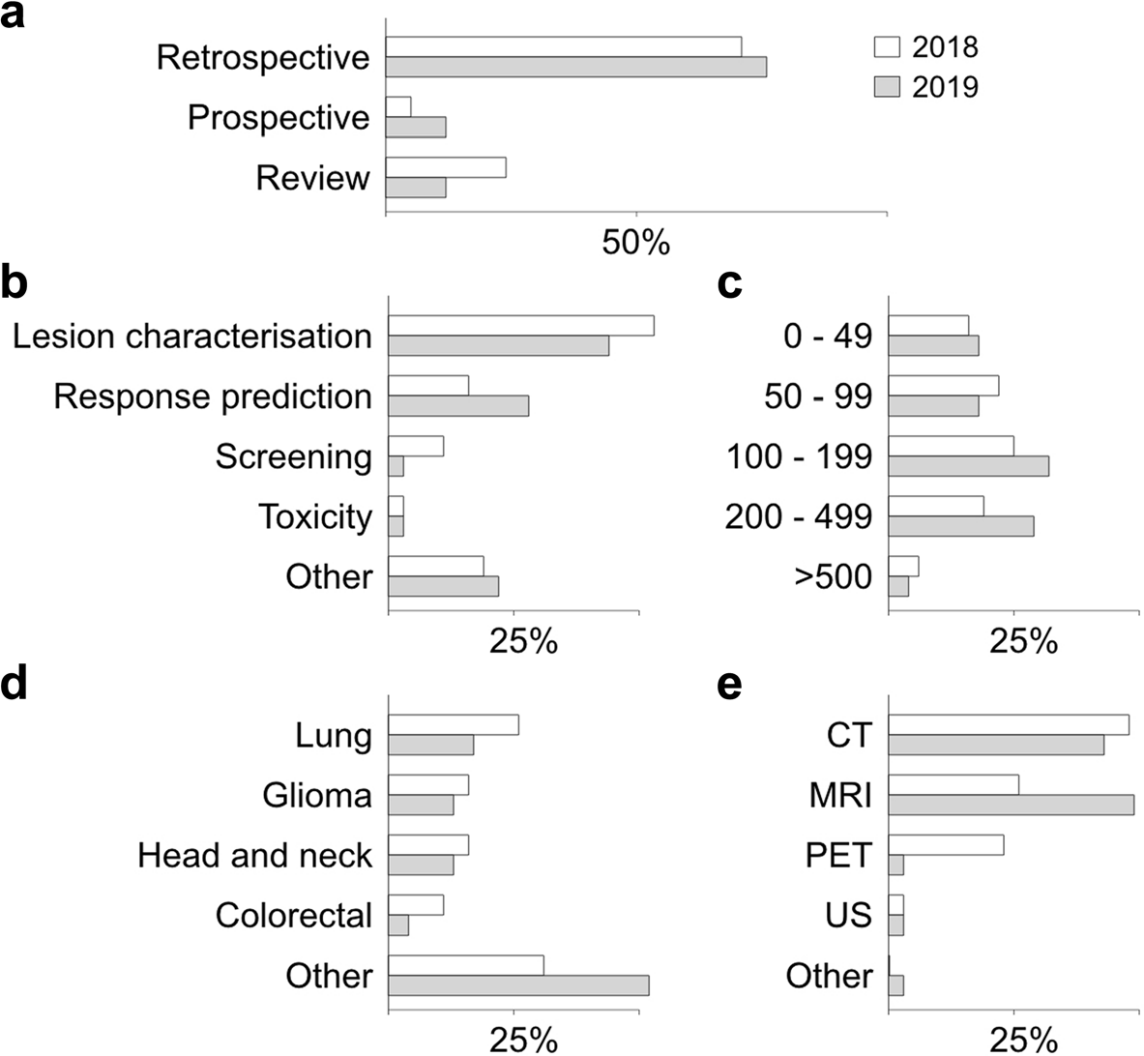
Research trends in radiomics from a sample of the first radiomics 40 papers of 2018 (white) and 2019 (grey) show a shift towards more diverse applications and larger cohorts of patients (see Additional file 1: Table S1 for PubMed query syntax). **a** Trial type. **b** Study aim. **c** Total size of cohort. **d** Disease type. **e** Imaging modality. *CT* Computed tomography, *MRI* Magnetic resonance imaging, *PET* Positron emission tomography, *US* Ultrasound

While radiomics make their way into clinical workflows, large or even dynamically growing prospective patient cohorts are required to reach the level of robustness and precision needed to be adopted by clinicians (Fig. 4). To better understand these challenges, it is important to grasp the workflow of radiomics, as well as the variety of algorithms and analytical models currently under development.

### Handcrafted radiomics techniques in radiomics research

The current state-of-the-art approach in radiomics relies on a relatively conventional image analysis workflow, which is referred to as “handcrafted” radiomics based on four successive processing tasks [5]: (1) image acquisition/reconstruction, (2) image segmentation, (3) feature extraction and quantification, and (4) feature selection/statistical analysis. In handcrafted radiomics, image features are known and are selected depending on their correlation with clinical outcome.

Image acquisition (step 1) and image segmentation (step 2) have been a lesser focus of radiomics publications. However, the risk of bias and potential lack of reproducibility of algorithms across different machines, protocols, and sites are well recognised [6–9] and standardisation issues are further discussed in the next section.

Feature extraction (step 3) in handcrafted radiomics has established several techniques based, for example, on image morphology, histograms, textures, wavelet, or fractal techniques, with their application in original works reviewed in detail by Avanzo et al. [10]. In the same review, the authors showed that statistical analysis/feature selection (step 4) has not reached a disease-specific or technical consensus on feature selection strategies, predictive models and performance estimation techniques. Linear regression models were shown to be more frequently used, which can be explained by more simple models providing graphical results (nomograms) [11], and a similar trend is noted in a 2019 sample of publications analysed (Additional file 1: Table S2). Avanzo et al. [10] further identified limitations of radiomics related to the size of the cohorts, and issues of standardisation and benchmarking, which are discussed in the next paragraphs.

### The emerging role of deep learning

Deep learning radiomics (DLR) can be applied to any aspect of the workflow described above. Many studies use DLR to either automatically identify or extract features (step 3). In other instances, a classical handcrafted radiomics approach extracts features so that a DLR algorithm can select them (step 4). Single artificial neural networks (ANN) can perform both tasks [5].

DLR is based on a subtype of machine learning technique based on ANN with a high number of interconnected layers. Given a training set, such networks can autonomously build image filters and extract image features for classification without the need to pre-determine (handcraft) them. In the field of medical imaging, several applications have already been successfully designed, for example, in detecting lung nodules [12] or in building computed tomography images from magnetic resonance images for the purpose of positron-emission tomography (PET) attenuation correction [13].

These multi-layer ANN are often convolutional (cANN) or recurrent (rANN) and, after training, produce image filters and features in a much higher number than other techniques. The major challenge of deep learning algorithms in radiomics is however the need for a higher number of observations (patients) than in many handcrafted radiomics studies.

However, once trained, a deep learning algorithm can be extremely fast and accurate [5]. Recently, Xu et al. [14] have developed a model based on a cANN and a rANN for overall survival prognosis in non-small cell lung cancer using a seed point tumour localisation instead of a classical segmented contour. The authors analysed how the tumour evolved during the time, exploiting several successive computed tomography scans acquired at different time points (pre-treatment, 1, 3, and 6 months after radiation therapy). Algorithms that can include multiple imaging time points are a key step to bring radiomics to the bedside and into personalised oncology data streams.

## Protocol standardisation in radiomics feature extraction

A fundamental aspect of radiomics is the capability of extracting quantitative image features (QIFs). QIFs are measures computed directly from the voxel values (intensity) and their spatial organisation (shape and texture). When compared to human-based visual analysis and semantic (*i.e.*, text) features, the main advantage of QIFs is to yield more objective and reproducible image analysis. However, this advantage comes with the following two challenges.

First, small changes in voxel values can lead to strong variation in values of QIFs, which in turn can modify the output of the associated predictive model and lead to inadequate classification.

Second, the definition, implementation and semantic grouping of QIFs can vary between scanners, studies, and software, which hinders the interoperability of QIFs and their interpretation [15]. These aspects are of particular importance for the integration of radiomics in clinical workflows. Both individual patients and patient cohorts may be managed in multiple centres and may benefit from medical imaging from different scanners and different imaging protocols.

The impact and potential solution to address the two aforementioned challenges are hence of particular interest.

### Standardisation of feature definition

The very first aspect of standardisation is to establish a common reference of the definitions of every QIFs. Second, it must be ensured that distinct software implementation of the same QIF will provide the same measure for a given input. To address these two aspects, the Image Biomarker Standardisation Initiative (IBSI) did a remarkable and exhaustive effort, where the QIF definitions are organised in a reference manual [6]. In addition, the IBSI defined a systematic benchmarking of radiomics software using synthetic and patient-based reference images, which allowed to achieve consensus for most QIFs, but often after several iterations of discussions, debug and re-implementation, showcasing the importance of the initiative. The description and benchmarking were not limited to QIFs, but also to image pre-processing and interpolation steps. Since most radiomics software and researchers joined the IBSI since September 2016, it will have a significant impact on the reproducibility of future radiomics studies and the interoperability of QIF extraction algorithms.

### Impact of imaging protocol on QIFs: towards standardisation and invariance

Imaging protocols relate to image acquisition and reconstruction and are depending on many factors including disease, anatomical regions and manufacturers and user preferences. Expert image readers are able to quickly adapt to changes in image quality and reconstruction parameters. However, because QIFs are computed from raw voxel values, changing acquisition protocols will have a direct influence on the extracted quantitative measures. This can be simply illustrated with the maximum standardised uptake value (SUV_max_), which measures the maximum uptake in PET imaging and was identified in many radiomics studies as an important biomarker [16, 17]. SUV_max_ will vary significantly based on the post-reconstruction smoothing of PET images (*e.g.*, Gaussian filtering), which deteriorates the generalisation performance of a SUV_max_-based radiomics model between imaging centres using different reconstruction protocols. Although achieving consensus protocols across all imaging departments and manufacturers is still unrealistic today, systematic studies of their impact on feature values are crucial to determine the deployment success of radiomics-based clinical models.

Traverso et al. [18] reviewed 41 studies focusing on repeatability and reproducibility of QIFs. Repeatability is defined as QIF stability when imaging the same subject (or phantom) over time and is often associated with the test-retest methodology [19]. Reproducibility addresses the stability of QIFs across imaging conditions (protocol, manufacturer) and is therefore more general than repeatability. The survey revealed that although no consensus could be found regarding the most repeatable and reproducible features, intensity (also called first-order) features were in general more reproducible than shape and textural QIFs.

As a further step towards reproducibility, several efforts focused on developing QIF transformations to achieve invariance to imaging protocols. A notable example is the use of the Combat method (initially developed in the context of genomics to remove machine and time variability in microarray data using Bayesian statistics [20]) to successfully standardise PET radiomics [21]. Neural networks and deep learning techniques such as *generative adversarial networks* were used in [22] to learn QIF transforms that makes them independent to scanner manufacturers and protocols. The transforms were learnt based on the Credence Cartridge Radiomics (CCR) physical phantom and associated dataset resulting from scanning with various imaging conditions. This radiomics-specific imaging phantom is made of 10 cartridges filled with various materials leading to a wide range of values of radiomics features [23].

## Integration of omics data with radiomics algorithms

While a definitive proof that combining all omics information can result in better precision in predicting clinical outcome is not easily achievable, it is important to note that this approach is commonly used in patient management: physicians gather available information from various diagnostic and examination techniques prior to devising a specific patient management plan.

Similarly, radiomics has witnessed an evolution from an initial focus on lesion identification (such as malignant *versus* non-malignant lesions) and prognosis (*i.e.*, assessing disease but not therapy). In more recent algorithms, genomic or immunomic features are used to address treatment selection or response (*i.e.*, predictive rather than prognostic) and to improve prediction of clinical outcomes (such as overall survival or toxicity) [24, 25]. These approaches can be described specifically as radiogenomics, respectively radioimmunomics. Furthermore, the term holomics is used to refer to more ambitious strategies where radiomics is one component of all “omics” axes used for clinical management in precision medicine strategies [26].

### Radiogenomics

The pioneer work of Gevaert et al. in 2012 opened the field of radiogenomic by studying the correlation of gene clusters with radiomics features in 26 non-small cell lung cancer [27]. The following year, Aerts et al. published one of the earliest studies correlated with genetic information [28]. In this milestone publication, the inclusion of more than 1,000 patients with lung or head and neck cancer led to a four-feature prognostic score. Interestingly, prognostic capability of the signature was maintained between tumour subtypes and in particular regardless of human papillomavirus (HPV) status of head and neck tumours. Gene-set enrichment analysis was used to correlate (and not predict) each feature with gene expression, inferring for example a relationship between tumour heterogeneity on imaging and cellular proliferation.

More recently, Digumarthy et al. showed that the combination of clinical, imaging and radiomics features can separate epidermal growth factor receptor mutant from wild-type lung adenocarcinoma with high accuracy [29]. The technical survey published in 2017 by Incoronato et al. explored the status of radiomics research on the correlation and/or prediction of genomic characteristics in 55 original research publications [25]. Incoronato et al. showed that correlation models were twice most frequent and that they were heavily reliant on statistical methods rather than machine learning with a majority of canonical standard models (Wilcoxon, chi-squared, Fisher’s test, Kruskal-Wallis), of canonical correlation techniques (Spearman, Pearson) and linear-regression based techniques (linear-regression, logistic regression, etc.). Deep learning approaches have also shown successes in predicting gene expression and mutation status in gliomas [30–32].

This field remains in constant expansion and further reviews such as Pinker et al. and El Naqa et al. analyse the current state of the art in multiple cancers combining radiomics with single nucleotide polymorphisms, copy number variations and gene expression amongst others [33, 34].

### Radioimmunomics

Immune checkpoint modulation and other immunotherapies have significantly increased therapeutic options in oncology and lead to a need for better predictive tools for therapy management decisions and better response follow-up [35]. The recent field of radioimmunomics is expected to further develop in the future. In current publications, the work of Sun et al. is of particular interest. The team developed a predictive radiomics model focusing of immunomics (CD8, cell infiltration in solid tumours) and subsequent response to immune checkpoint therapy (anti-PD-1 or anti-PD-L1) [36]. Interestingly, this retrospective work on four cohorts of above 100 patients each did not restrict itself to a single tumour histology but used databases of patients with solid tumours. It used computed tomography images to build a radiomics biomarker of CD8 cells infiltration (using transcription of CD8B ribonucleic acid in biopsy samples) which showed prediction power for response to immunotherapy. This handcrafted radiomics study was based on machine learning (not deep learning) using a linear elastic-net model with 78 radiomics and six additional features (peak voltage and five binary location variables).

### Data heterogeneity in holomics

The challenge of data heterogeneity becomes critical when combining radiomics approaches with genomic or immunomic data or when building holomics workflows. The data contained in an imaging study (voxels values, protocols, series, etc.) is neither numerical (*i.e.*, a single continuous number) nor categorical (*i.e.*, positive *versus* negative, low *versus* moderate *versus* high). Many radiomics strategies are based on statistical methods in order to reduce the dimensionality of image data by focusing on individual continuous numerical variables (*i.e.*, the features themselves). In deep learning, popular techniques such as convolutional ANN (cANN) are able to acquire in input the whole image, exploit the informative content of the pixel/voxel proximity, and automatically build representative image features and filters to reduce the noise due to signal ratio. However, cANN have been developed specifically for images and incorporating individual numerical or categorical variables (ECG signals, lab tests, pathology results) adds further complexity. More recently, ensemble learning has been introduced to combine several predictors that use different data types [37, 38]. Ensemble learning is widely adopted in other domains (*e.g.*, information retrieval, where a meta-predictor is built by voting on different predictors [39]).

## Big data challenges for precision medicine

### Challenges of health and imaging biobanks

The developments of imaging biobanks and cloud-based data storage services have radically changed the way in which communication and data management are dealt with. Online storage of medical images is not new; several vendors have offered such services for decades already as part of their commercial solutions [39, 40]. Recently, a major change is the emergence of widely accessible and attractive services at a very low cost. These developments face more hurdles in medical applications, due to strict regulations and guidelines for patient confidentiality and data security.

The wealth of data acquired in clinical routine these days is overwhelming and has not been apprehended yet; medical images are no exception.

A crucial limiting factor in the development of radiomics for large and open cohorts in order to be applicable in clinical workflows is the lack of open and collaborative infrastructure collecting constantly structured and well-documented imaging data [41]. Restrictive regulatory constraints and data protection rules further prevent the usage and exploitation of medical data without formal patient approval. Overall, the walls between medical institutions, medical records, and all clinical data repositories have remained despite multiple initiatives from individual institutions, professional societies, and private corporations to facilitate the accessibility to the data. Data extraction and anonymisation for open-access databanks is indeed still very limited [42].

Recent years have seen the development of international consortia and networks to facilitate sample collection and distribution to allow access to precious biomaterials and medical data from patients [43]. Such repositories should provide open access to carefully curated and annotated data. These initiatives however face many challenges that have been long-recognised and theorised [44, 45]. He identified the key restrictions to the development of an open repository to harbour radiomics tools and computer-aided personalised medicine.

Despite these initiatives, many hurdles remain. Data collection is complex due to institutional, infrastructure and regulatory issues. Further, ownership of data by the source institution and the patient is not guaranteed from data collection to utilisation, in particular as data itself may be considered the source of value. This issue is a major difficulty to reach broad acceptance and clinical application. We hypothesise that it may be circumvented by adopting open source and open data architectures as these systems benefit from wide acceptance. Finally, as databases grow, software tools that incorporate new data need to be developed, in particular based on deep learning approaches within environments that facilitate software development.

### Initiatives for precision medicine

In parallel, there is a significant attempt by national authorities and private initiatives to channel the amount of data to better predict disease occurrence and behaviour, guide therapy management, and predict outcome.

Amongst these, notably the National Institute for Health (NIH) has launched the “All of Us” research programme aiming at accumulating data on over a million participants. Further research initiatives in the USA towards precision medicine in oncology have been developed in the past decade by the National Cancer Institute. The NCI initiative has designated cancer imaging as one of the four principal axes of this initiative along translational research, genomics and immunomics.

Switzerland has initiated a nationwide initiative for the support and development of personalised medicine called the “Swiss Personalized Health Network” [46] aimed at the harmonisation and deployment of large networks of information technology infrastructures and data repository for future support of artificial intelligence and computer-aided medical decisions and personalised patient management. Notably, within this initiative, a global ethics approval across adopted by each Swiss university hospital is being implemented [47].

Amongst several initiatives currently underway to sustain the development of such networks, a new platform is being specifically designed for hosting and sharing medical imaging data for the implementation of machine learning and radiomics tools. This open platform, called KHEOPS [48], offers a flexible solution for storing, sharing and viewing medical images. KHEOPS is an open-source project with the source code available on GitHub which provides the framework for secure and flexible management of extensive collections of digital images in open and scalable infrastructure for storage, communication, wide distribution, processing, and analysis of this data. In an intermediate step to circumvent data ownership and privacy issues, initiatives to federate research centres around multiple data sources and data types (including images) are already established, such as VATE [49] and EuroCAT [50]. As opposed to KHEOPS, these are based on distributed learning, where each institution maintains a databank and only shares data aggregation.

## Conclusions

With the development of radiomics as a mainstream research field, major efforts have been undertaken in the development of new algorithms. In recent years, radiogenomics, radioimmunomics, or other clinical data have been used to increase predictive capacity and accuracy. Concerted efforts and initiatives aiming for data harmonisation between imaging protocols, scanners and imaging devices as well as quality control of algorithms being used for feature extraction are also well underway.

As the field has now matured, the shift from individual algorithms engineered (handcrafted) from finite datasets (*i.e.*, patient cohorts) to open tools that dynamically integrate the constant inflow of medical data is paramount. However, reaching this goal has so far remained elusive, despite many initiatives from institutions, professional societies, manufacturers, and major information technology and Internet corporations.

The time has hence come for a paradigm shift. The imaging and clinical databases can only thrive if data extraction itself is facilitated and if institutional and patient ownership is extended to every stage of data indexing, storage, and analysis. Further, mirroring the dynamic nature that should be adopted by these databases, radiomics, and other software tool development have to be facilitated. The whole environment must embrace full web application programming interfaces, dematerialised data storage, and provide persistent and secure data ownership. We believe that such infrastructure can be achieved with the confidence that offers open source and open data technologies.

It will provide the backbone for thriving, evolutive and collaborative radiomics and holomics tools to ultimately reach clinical workflows. The medical imaging community has the opportunity and responsibility to lead these developments in order for precision medicine based on comprehensive holomics computer-assisted expert systems to mediate a revolution in medical practice and patient management.

## Supplementary information

**Additional file 1: Table S1.** Detailed results and syntax of PubMed “radiomics” search. **Table S2.** Occurrence and category of statistic techniques in radiomics from an arbitrary sample of the 40 first research papers referenced in PubMed in 2019.

## Data Availability

Not applicable

## Abbreviations

ANN: Artificial neural network
cANN: Convolutional artificial neural network
CD8: Cluster of differentiation 8 (CD8 cells, cytotoxic lymphocytes)
DLR: Deep learning radiomics
PD-1: Programmed death 1
PD-L1: Programmed death-ligand 1
PET: Positron emission tomography
QIF: Quantitative image features
rANN: Recurrent artificial neural network
SUV_max_: Maximum standardised uptake value
