# Supplementary material for: Integrating radiomics into holomics for personalised oncology: from algorithms to bedside
Source: Eur Radiol Exp. 2020 Feb 7;4:11. doi: 10.1186/s41747-019-0143-0 (PMC7007467; doi:10.1186/s41747-019-0143-0)

**Table S1.** Detailed results and syntax of PubMed “radiomics” search.


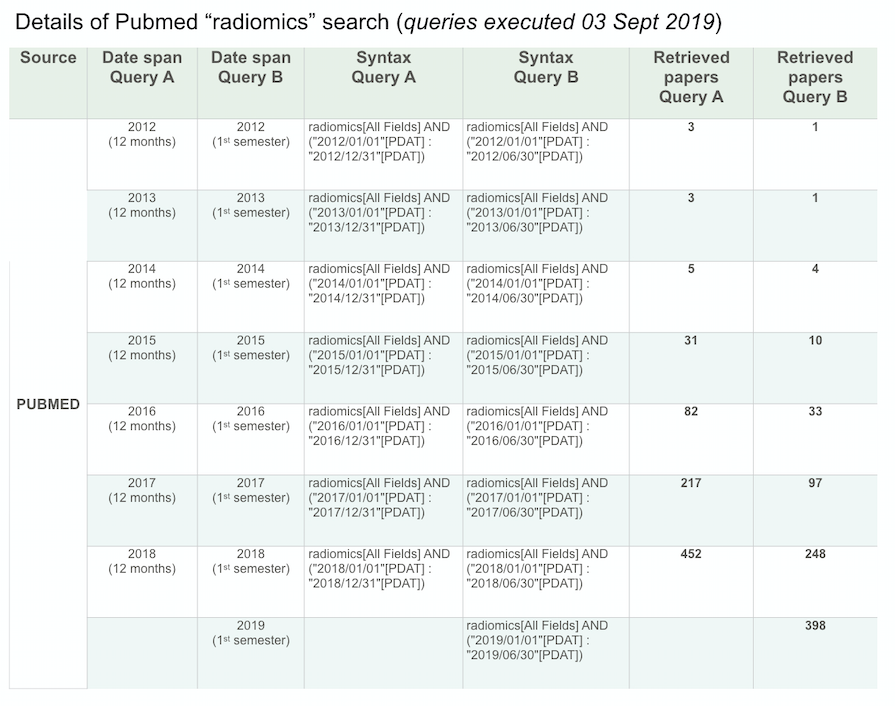


**Table S2.** Occurrence and category of statistic techniques in radiomics from an arbitrary sample of the 40 first research papers referenced in PubMed in 2019.


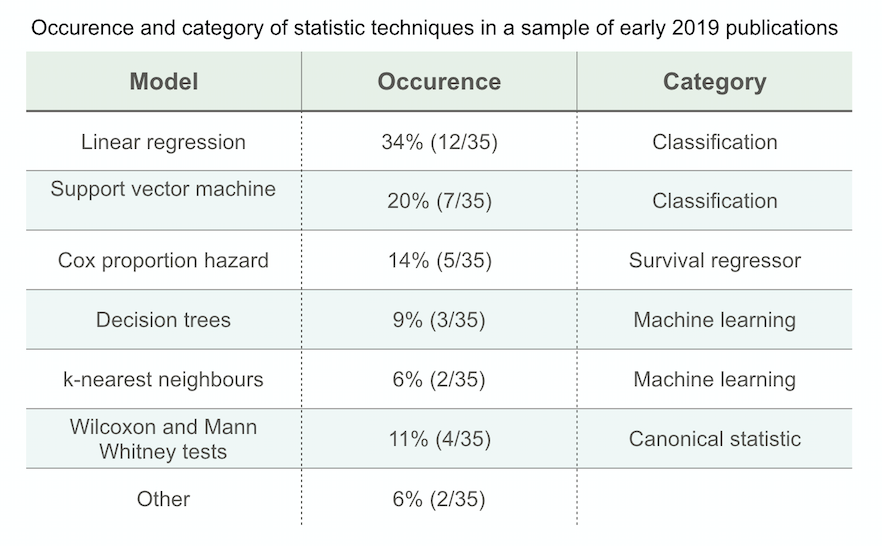

Supplement: Supplementary file 1 — Additional file 1: Table S1. Detailed results and syntax of PubMed “radiomics” search. Table S2. Occurrence and category of statistic techniques in radiomics from an arbitrary sample of the 40 first research papers referenced in PubMed in 2019. [file 41747_2019_143_MOESM1_ESM.docx]
